# Outcomes and Predictive Factors for Gynecologic Organ Involvement in Radical Cystectomy for Female Bladder Cancer Patients: A Multicenter Retrospective Cohort Study

**DOI:** 10.3390/medicina62081609

**Published:** 2026-08-21

**Authors:** Mehmet Gürkan Arikan, Ersan Arda, Volkan İzol, Hasan Yilmaz, Evren Suer, Murat Akgul, Sertac Yazici, Deniz Bolat, Guven Aslan, Serkan Akan, Levent Turkeri

**Affiliations:** 1 Department of Urology, School of Medicine, Çukurova University, Adana 01330, Türkiye; 2 Department of Urology, School of Medicine, Trakya University, Edirne 22030, Türkiye; 3 Department of Urology, Çukurova Urology Center, Adana 01170, Türkiye; 4 Department of Urology, School of Medicine, Kocaeli University, Kocaeli 41001, Türkiye; 5 Department of Urology, School of Medicine, Ankara University, Ankara 06230, Türkiye; 6 Department of Urology, Ümraniye Training and Research Hospital, University of Health Sciences, Istanbul 34668, Türkiye; 7 Department of Urology, School of Medicine, Hacettepe University, Ankara 06800, Türkiye; 8 Department of Urology, İzmir City Hospital, İzmir 35540, Türkiye; 9 Department of Urology, School of Medicine, Dokuz Eylül University, İzmir 35220, Türkiye; 10 Department of Urology, Fatih Sultan Mehmet Training and Research Hospital, Istanbul 34752, Türkiye; 11 Department of Urology, Acıbadem Mehmet Ali Aydınlar University Hospital, Istanbul 34638, Türkiye

**Keywords:** radical cystectomy, muscle-invasive bladder cancer, pathological gynecologic organ involvement, organ preservation, survival outcomes

## Abstract

*Background and Objectives*: Radical cystectomy (RC) is standard treatment for muscle-invasive bladder cancer, but the role of gynecologic organ preservation in women remains uncertain. We primarily evaluated the prevalence and predictors of pathological gynecologic organ involvement and secondarily assessed survival and developed a prediction model. *Materials and Methods*: This multicenter retrospective study included 232 women undergoing RC at 13 tertiary centers. Of the 232 women, 182 had clinical T2 disease and 50 had BCG-unresponsive or very-high-risk Ta/T1 non-muscle-invasive bladder cancer. Overall survival (OS) was the primary endpoint; CSS and RFS were secondary outcomes. Multivariable logistic regression was used to identify predictors and develop preoperative prediction models. *Results*: Pathological gynecologic organ involvement was identified in 26 patients (11.2%): uterine in 15 (6.5%), vaginal in 11 (4.7%), ovarian in five (2.2%), and adnexal in 21 (9.1%). The median OS was shorter in patients with involvement than in those without (19.0 vs. 48.0 months; *p* = 0.048). Vaginal and ovarian involvement were associated with shorter OS (both *p* < 0.001), whereas uterine and adnexal involvement were not. Pathological T stage was associated with organ involvement (*p* = 0.001), with the highest frequency in pT4 disease. Model 2 showed an AUC of 0.898 and an optimism-corrected AUC of 0.872. A risk score of 0 classified 31.0% as low risk, with an NPV of 97.2% and a sensitivity of 92.3%. *Conclusions*: Pathological gynecologic organ involvement was uncommon but associated with poorer survival. These findings may support preoperative risk stratification, although the oncologic safety of organ-preserving surgery cannot be established from pathological involvement data alone.

## 1. Introduction

Bladder cancer (BC) is the eleventh most common malignancy in women and the tenth most common malignancy globally, with approximately 573,000 new cases and 213,000 deaths annually [1,2]. Muscle-invasive BC (MIBC) represents a particularly aggressive subset of BC, constituting approximately 20 to 30% of all BC cases [2]. In women, MIBC is associated with poor prognosis, with five-year survival rates of only 30 to 40% despite aggressive treatment approaches [3,4,5]. Radical cystectomy (RC) remains a standard curative treatment for non-metastatic and an important treatment option for BCG-unresponsive and very-high-risk non-muscle-invasive bladder cancer [6,7]. However, RC in women frequently requires extensive resection of adjacent gynecologic organs, including the uterus, Fallopian tubes, ovaries, and anterior vaginal wall, as part of anterior pelvic exenteration to ensure negative surgical margins [8,9,10]. This comprehensive approach is critical for minimizing the risk of local recurrence, particularly in cases of high-grade histology, advanced T stage, or lymphovascular invasion (LVI) [11,12].

While the oncologic benefits of RC are well established, the extensive nature of this surgery can lead to significant postoperative morbidities, particularly affecting urinary, sexual, and hormonal functions. Several studies have shown that up to 70% of female patients experience postoperative sexual dysfunction, with additional risks of hormonal imbalances that can lead to osteoporosis and cardiovascular disease due to oophorectomy [13,14,15]. The potential for pelvic organ prolapse further contributes to the decline in quality of life, emphasizing the need for approaches that balance oncologic safety with functional outcomes [15].

Recent advancements in imaging, a deeper understanding of pelvic organ functions, and improved surgical techniques have spurred interest in gynecologic organ-sparing RC (GO-SRC) [9,12,16,17]. Organ-sparing techniques focus on preserving critical structures, such as the neurovascular bundle, uterus, ovaries, and portions of the vaginal wall, particularly in cases of organ-confined (≤T2) disease with no direct involvement of these organs. The feasibility of GO-SRC without compromising oncologic outcomes has been increasingly investigated, supported by evidence suggesting that pathological gynecologic organ involvement in RC specimens is relatively rare. However, the absence of histopathological involvement does not demonstrate the oncologic safety of organ preservation. Although these concepts are related, the oncologic safety of organ-preserving surgery additionally depends on surgical margin status, occult disease, recurrence patterns, and long-term cancer outcomes [10,16,17,18].

Previous studies have identified several clinicopathological features associated with gynecologic organ involvement, including locally advanced tumor stage, lymphovascular invasion, tumor location at the bladder neck or trigone, preoperative hydronephrosis, variant histology, lymph node involvement, tumor size, and concomitant carcinoma in situ [9,11,19,20]. However, the reported predictors have varied across studies, and most available evidence has been derived from retrospective cohorts with limited sample sizes. Therefore, a parsimonious preoperative risk stratification approach based on routinely available clinical variables remains necessary. In this context, this multicenter study sought to identify factors associated with pathological gynecologic organ involvement and to develop and internally validate a reduced preoperative prediction model to facilitate individualized preoperative risk assessment.

The European Association of Urology (EAU) and American Urological Association (AUA) guidelines do not recommend routine organ preservation during RC but acknowledge its potential applicability in select cases where organ preservation may not compromise oncologic control [10,12,18]. Therefore, this study’s primary aim was to determine the prevalence of histopathologically confirmed pathological gynecologic organ involvement and to identify preoperative factors associated with such involvement in women undergoing RC for bladder cancer. The secondary aims were to evaluate the association between pathological gynecologic organ involvement and survival outcomes and to develop and internally validate a preoperative prediction model to support risk stratification and individualized surgical planning.

## 2. Materials and Methods

### 2.1. Study Design and Ethical Framework

This multicenter, retrospective cohort study included female patients who underwent radical cystectomy between July 2004 and October 2023. Data were retrospectively retrieved from the Bladder Cancer Database of the Turkish Urooncology Association, which includes patients treated at 13 high-volume tertiary referral centers specializing in urologic oncology.

**Figure 1:**
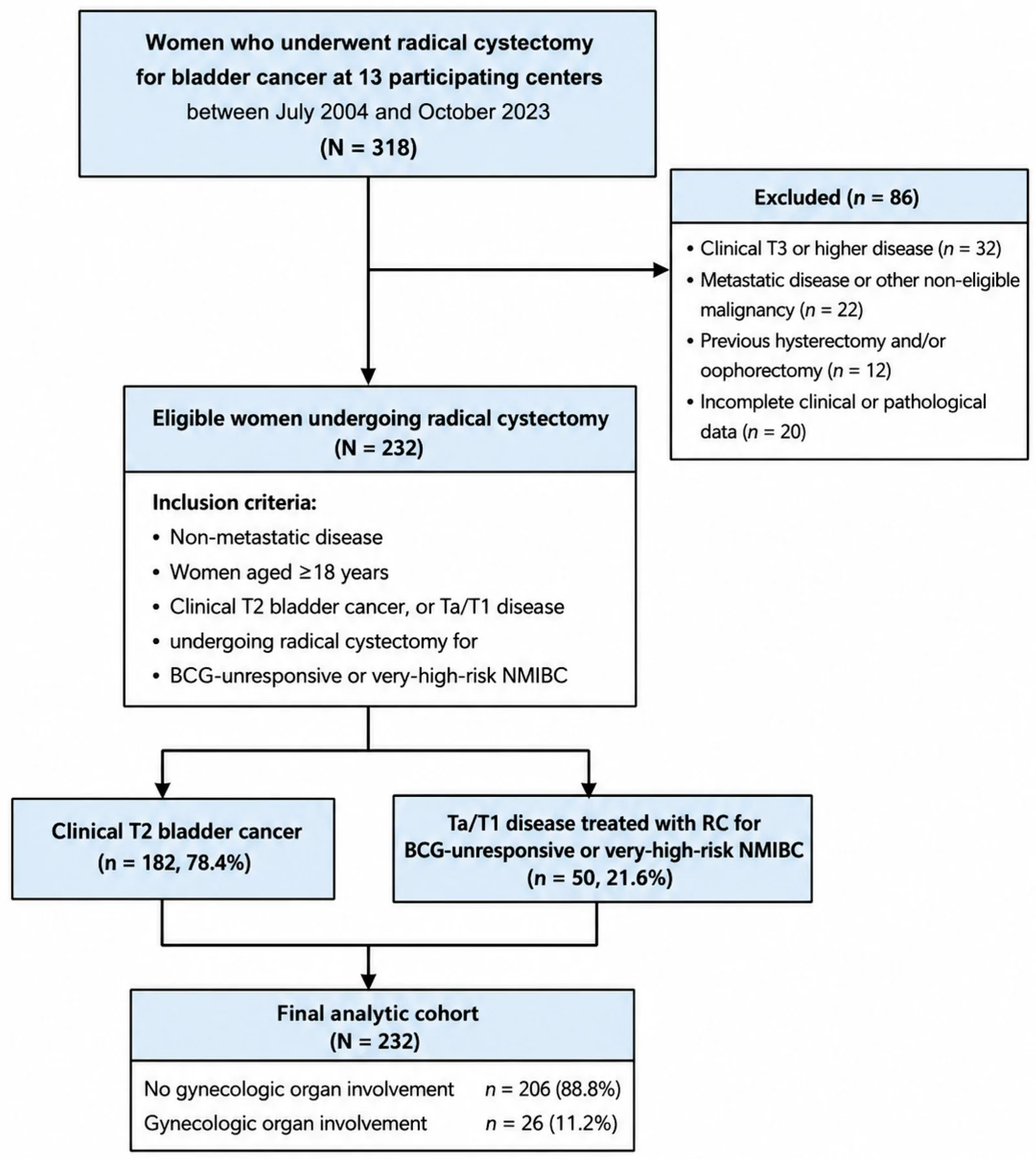
Patient selection flowchart. BCG, Bacillus Calmette–Guérin; NMIBC, non-muscle-invasive bladder cancer; RC, radical cystectomy.

Eligible patients included women aged ≥ 18 years with non-metastatic bladder cancer who underwent RC with curative intent. The cohort included patients with clinical T2 bladder cancer and selected patients with Ta/T1 disease who underwent RC for BCG-unresponsive or very-high-risk non-muscle-invasive bladder cancer. Patients with clinical T3 or higher disease, metastatic disease at diagnosis, previous hysterectomy and/or oophorectomy, previous or concomitant gynecologic malignancy or other non-bladder primary malignancy, or incomplete clinical or pathological data required for the primary analyses were excluded. A total of 318 women were screened, of whom 86 were excluded according to the predefined criteria, leaving 232 women in the final analytic cohort. The patient selection process is summarized in Figure 1. Eligibility was based on the preoperative clinical/TUR-based disease category. The pathological T stage reported in Table 1 was determined from the final radical cystectomy specimen and was not used as an inclusion criterion. Patients were included regardless of whether they received neoadjuvant chemotherapy.

This study was approved by the Institutional Review Boards of all the participating centers, as required (no.: 2026/165-29; date: 3 April 2026). Written informed consent was obtained from each patient for all diagnostic and therapeutic procedures. This study was conducted in accordance with the principles of the Declaration of Helsinki.

### 2.2. Surgical Technique

All RC procedures were performed by experienced urologic surgeons using standard techniques for anterior pelvic exenteration at the participating centers. In all patients included in the analytic cohort, RC included removal of the bladder together with the uterus, ovaries, Fallopian tubes, and anterior vaginal wall as part of anterior pelvic exenteration. These gynecologic structures were pathologically evaluated in the final cystectomy specimen, and organ-specific involvement was recorded. Accordingly, the entire analytic cohort (*n* = 232) was used as the denominator for organ-specific pathological involvement frequencies.

### 2.3. Data Collection and Outcome Measures

Clinical, demographic, surgical, and pathological data of the patients, including age, body mass index (BMI), smoking status, comorbidities, neoadjuvant chemotherapy, and surgical approach, were recorded. Initially incomplete clinicopathological records were retrospectively re-reviewed in the source database before the final analyses to ensure data completeness. Tumor-related variables included transurethral resection (TUR) pathology, tumor size, multifocality, preoperative hydronephrosis, clinical and pathological TNM stage, tumor grade, LVI, perineural invasion, carcinoma in situ, and histological subtype. Histological findings included conventional urothelial carcinoma, divergent differentiation, and variant histology, including sarcomatoid, micropapillary, plasmacytoid, and other recognized variants.

The primary outcome was histopathologically confirmed involvement of one or more gynecologic organs, including the uterus, ovaries, Fallopian tubes, adnexa, or vaginal wall. Cancer-specific survival (CSS) was defined as the time from radical cystectomy to death attributable to bladder cancer, with deaths from other causes censored. Recurrence-free survival (RFS) was defined as the time from radical cystectomy to the first documented pelvic or systemic recurrence, with patients without recurrence censored at the last follow-up. CSS and RFS were evaluated as secondary survival outcomes. Survival probabilities at key time points—12, 24, 36, 48, and 60 months—were estimated.

A standardized organ-specific assessment of pathological gynecologic organ involvement on preoperative imaging at initial diagnosis was not systematically available in the retrospective database; therefore, preoperative clinical gynecologic organ involvement could not be reliably analyzed. The study outcome was based on histopathologically confirmed involvement in the radical cystectomy specimen. Throughout this manuscript, pathological gynecologic organ involvement refers to this histopathologically confirmed endpoint, whereas preoperative imaging-based assessment is referred to as clinical gynecologic organ involvement.

Postoperative follow-up was performed according to the routine protocols of the participating centers and was not fully standardized across institutions. Follow-up assessments included clinical evaluation and radiological imaging, with additional investigations performed when clinically indicated. The timing and frequency of surveillance evaluations varied across centers and over the study period. Recurrence and survival status were determined from institutional follow-up records.

### 2.4. Pathological Examination

The RC specimens were evaluated by dedicated genitourinary pathologists at the participating centers. Histopathological examination included assessment of the bladder tumor, surgical margins, lymph nodes, and the resected gynecologic organs. Histopathologically confirmed tumor involvement of the uterus, ovaries, Fallopian tubes, adnexa, or vaginal wall was recorded separately from the pathological T category of the bladder primary. The pathological T stage reported in Table 1 refers to the final pathological stage in the radical cystectomy specimen, whereas TUR pathology refers to the preoperative transurethral resection specimen. Direct invasion of the uterus or vagina was classified as pT4a according to the TNM system. Pathological T and N stages were assigned according to the applicable TNM classification system. Variant histology, divergent differentiation, LVI, perineural invasion, and soft-tissue involvement were also documented.

### 2.5. Statistical Analysis

Statistical analysis was performed using the IBM SPSS for Windows version 25.0 software (IBM Corp., Armonk, NY, USA). Continuous variables were presented as means ± standard deviation (SD) or medians and interquartile ranges (IQRs), while categorical variables were presented as numbers and frequencies. Given the skewed distribution of follow-up duration and time to recurrence, these variables were summarized using the median and interquartile range (IQR). Continuous variables were analyzed using Student’s *t*-test or the Mann–Whitney U test, while categorical variables were analyzed using the chi-square test or Fisher’s exact test. Univariate and multivariate binary logistic regression analyses were performed to identify predictors of pathological gynecologic organ involvement, and the results were reported as odds ratios with 95% confidence intervals (CIs). Three models of increasing complexity were evaluated. Model 1 included T2 disease on TUR pathology, with Ta/T1 as the reference category, and tumor size > 3 cm. Ta/T1 was used as the reference category because these patients represented the lower-stage TUR pathology subgroup within the analytic cohort and had undergone RC for BCG-unresponsive or very-high-risk NMIBC. Pathological T stage reported in Table 1 was evaluated separately and was not included in the preoperative prediction models, which used TUR pathology T2, with Ta/T1 as the reference category. Model 2 additionally included preoperative hydronephrosis and was designated as the primary reduced preoperative model, as it comprised routinely available clinical variables while maintaining model parsimony. Model 3 additionally included multifocal tumor and variant histology in the TUR specimen and was evaluated as an extended exploratory model. Model construction followed a hierarchical approach that prioritized routinely available preoperative variables and model parsimony. Given the limited number of pathological gynecologic organ involvement events (*n* = 26), Model 2 was retained as the primary prediction model, whereas Model 3 and the broader eight-variable multivariable analysis presented in Table 2 and Table 3 were considered exploratory given their lower EPV and greater potential for overfitting. Given the low number of outcome events relative to the number of predictors in the eight-variable exploratory model, Firth penalized logistic regression was additionally performed as a sensitivity analysis to reduce small-sample bias and the potential instability of maximum-likelihood estimates. Penalized odds ratios with 95% confidence intervals were reported. Model performance was assessed in terms of discrimination, calibration, overall prediction error, model complexity, and multicollinearity. Discrimination was quantified using the area under the receiver operating characteristic curve (AUC) with the 95% CI. Internal validation and optimism correction were performed using bootstrap resampling. Calibration was evaluated using the calibration slope and the Hosmer–Lemeshow goodness-of-fit test, while overall prediction error was assessed using the Brier score. Sensitivity, specificity, positive predictive value (PPV), and negative predictive value (NPV) were calculated at the probability threshold identified using the Youden index. In a complementary descriptive analysis, a risk score of 0, corresponding to the absence of all predictors included in a given model, was used to define the low-risk group. The NPV and proportion of patients classified as low risk were subsequently calculated for each model.

Center-level clustering was considered; however, because only 26 pathological gynecologic organ involvement events were distributed across 13 participating centers, mixed-effects or center-clustered regression models were not fitted due to the risk of unstable estimates with sparse within-center event counts. Therefore, potential residual center-level heterogeneity was considered a study limitation.

OS was the principal survival endpoint and was estimated using the Kaplan–Meier method and compared between groups using the log-rank test. CSS and RFS were estimated separately using Kaplan–Meier methods as secondary survival outcomes. No formal statistical comparisons were performed among OS, CSS, and RFS because these represent correlated endpoints within the same cohort. Pairwise comparisons among mutually exclusive involvement groups were adjusted using the Bonferroni method. Breslow and Tarone–Ware tests were performed as sensitivity analyses. Univariate and multivariate Cox proportional hazards regression models were used to assess factors associated with OS, with the results reported as hazard ratios (HRs) and 95% CIs. The multivariable Cox model was intended as a parsimonious adjusted analysis based on selected covariates rather than as a fully specified causal model. Postoperative pathological factors were not comprehensively included; therefore, residual confounding by disease severity cannot be excluded. A post hoc calendar-era sensitivity analysis was performed to assess temporal heterogeneity. Patients were compared between the 2004–2013 and 2014–2023 periods, and calendar time was also evaluated continuously per 5-year increment. Fisher’s exact test and univariable logistic regression were used, as appropriate. A two-sided *p*-value of <0.05 was considered statistically significant.

## 3. Results

The patient selection process is shown in Figure 1. A total of 318 women were screened during the study period. After exclusion of 86 patients according to the predefined eligibility criteria, 232 women were included in the final analytic cohort. Of these, 182 (78.4%) had clinical T2 bladder cancer, and 50 (21.6%) underwent RC for BCG-unresponsive or very-high-risk Ta/T1 non-muscle-invasive bladder cancer. The mean age was 63.27 ± 10.09 years, and the mean BMI was 26.70 ± 5.03 kg/m^2^. Histopathologically confirmed gynecologic organ involvement was identified in 26 patients (11.2%). Because the relevant gynecologic structures were pathologically evaluated in all 232 patients, organ-specific frequencies were calculated using the entire analytic cohort as the denominator. Uterine involvement was identified in 15 patients (6.5%), vaginal involvement in 11 (4.7%), ovarian involvement in five (2.2%), and adnexal involvement in 21 (9.1%). Neoadjuvant chemotherapy was administered in 26 patients (11.2%). The surgical approach was open in 212 patients (91.4%) and laparoscopic/robot-assisted in 20 patients (8.6%). No gynecologic organ preservation was performed in the study cohort (0/232, 0%). All patients underwent anterior pelvic exenteration, including removal of the uterus, ovaries, Fallopian tubes, and anterior vaginal wall; therefore, no organ-specific preservation criteria were applied. Systemic recurrence occurred in 7.5% of cases, while pelvic recurrence was observed in 7.0%. Among the 231 patients included in the survival analyses, 98 deaths occurred, corresponding to an overall mortality rate of 42.4%. Pathological gynecologic organ involvement was observed in 33.3% of patients with the sarcomatoid variant and 28.6% of those with glandular differentiation; however, the overall association between variant histology and pathological gynecologic organ involvement was not statistically significant (*p* = 0.237) (Table 1).

In the calendar-era sensitivity analysis, all 232 patients were classified according to treatment era. Pathological gynecologic organ involvement occurred in 11 of 70 patients (15.7%) treated during 2004–2013 and in 15 of 162 patients (9.3%) treated during 2014–2023, with no statistically significant difference between eras (Fisher’s exact *p* = 0.180). In the univariable logistic regression, treatment during 2014–2023 was not significantly associated with pathological gynecologic organ involvement compared with 2004–2013 (OR = 0.55, 95% CI: 0.24–1.26; *p* = 0.180). Similarly, calendar time modeled continuously showed no significant temporal association (OR per 5-year increment = 0.93, 95% CI: 0.61–1.41; *p* = 0.738) (Supplementary Table S1).

**Figure 2:**
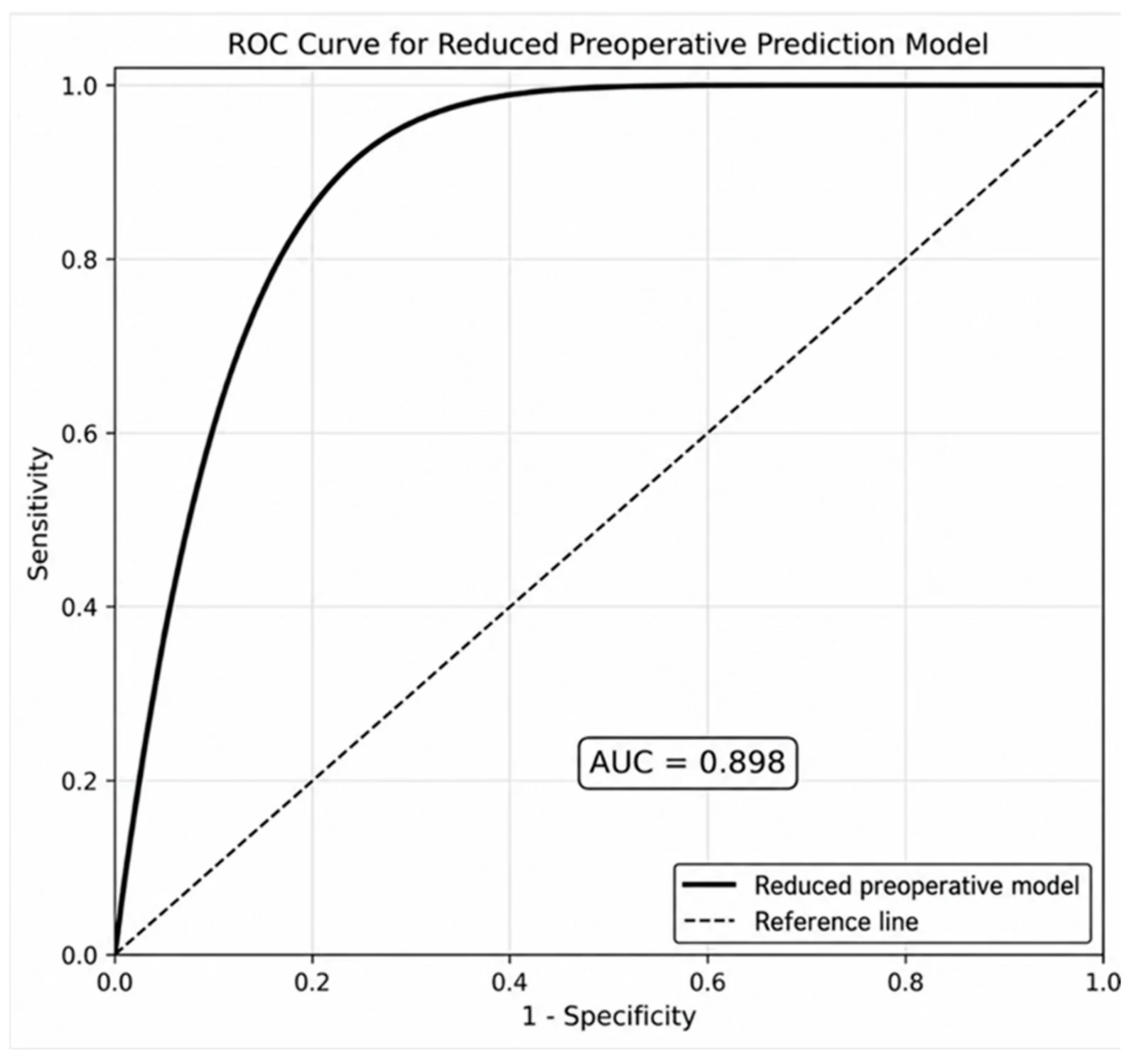
Receiver operating characteristic curve of the primary reduced preoperative prediction model for pathological gynecologic organ involvement. Model 2 included TUR pathology T2 (reference: Ta/T1), tumor size > 3 cm, and preoperative hydronephrosis. The AUC was 0.898.

Among the evaluated models, Model 3 achieved the highest apparent AUC; however, its lower events-per-variable ratio and calibration slope suggested a greater risk of overfitting. Model 2 demonstrated the most balanced overall performance, combining high discrimination, favorable optimism-corrected AUC, a low Brier score, acceptable calibration, and no relevant multicollinearity. Accordingly, Model 2 was selected as the primary preoperative prediction model, whereas Model 3 was retained as an extended exploratory model (Table 2 and Figure 2). The regression coefficients, intercept, and complete prediction equation for Model 2 are provided in Supplementary Table S2. Using a risk score of 0 to define low-risk status, Model 2 classified 31.0% of patients as low-risk and achieved a NPV of 97.2% with a sensitivity of 92.3% (Supplementary Table S3). For comparison, the overall probability of absence of pathological gynecologic organ involvement in the entire cohort was 88.8%. Thus, the Model 2 low-risk definition increased the NPV by 8.4 percentage points while classifying 31.0% of the cohort as low-risk; only two of 26 patients (7.7%) with pathological gynecologic organ involvement were classified in this low-risk group.

Tumor size > 3 cm, multifocality, preoperative hydronephrosis, and variant histology in the TUR specimen were statistically significantly associated with pathological gynecologic organ involvement after adjustment (Table 3). Given the low EPV (3.25), these estimates should be interpreted cautiously.

In the Firth penalized logistic regression sensitivity analysis, the direction and magnitude of the associations were generally consistent with the conventional multivariable model. TUR pathology T2, tumor size > 3 cm, preoperative hydronephrosis, and variant histology remained significantly associated with pathological gynecologic organ involvement, whereas the association with multifocality was attenuated (Supplementary Table S4).

**Figure 3:**
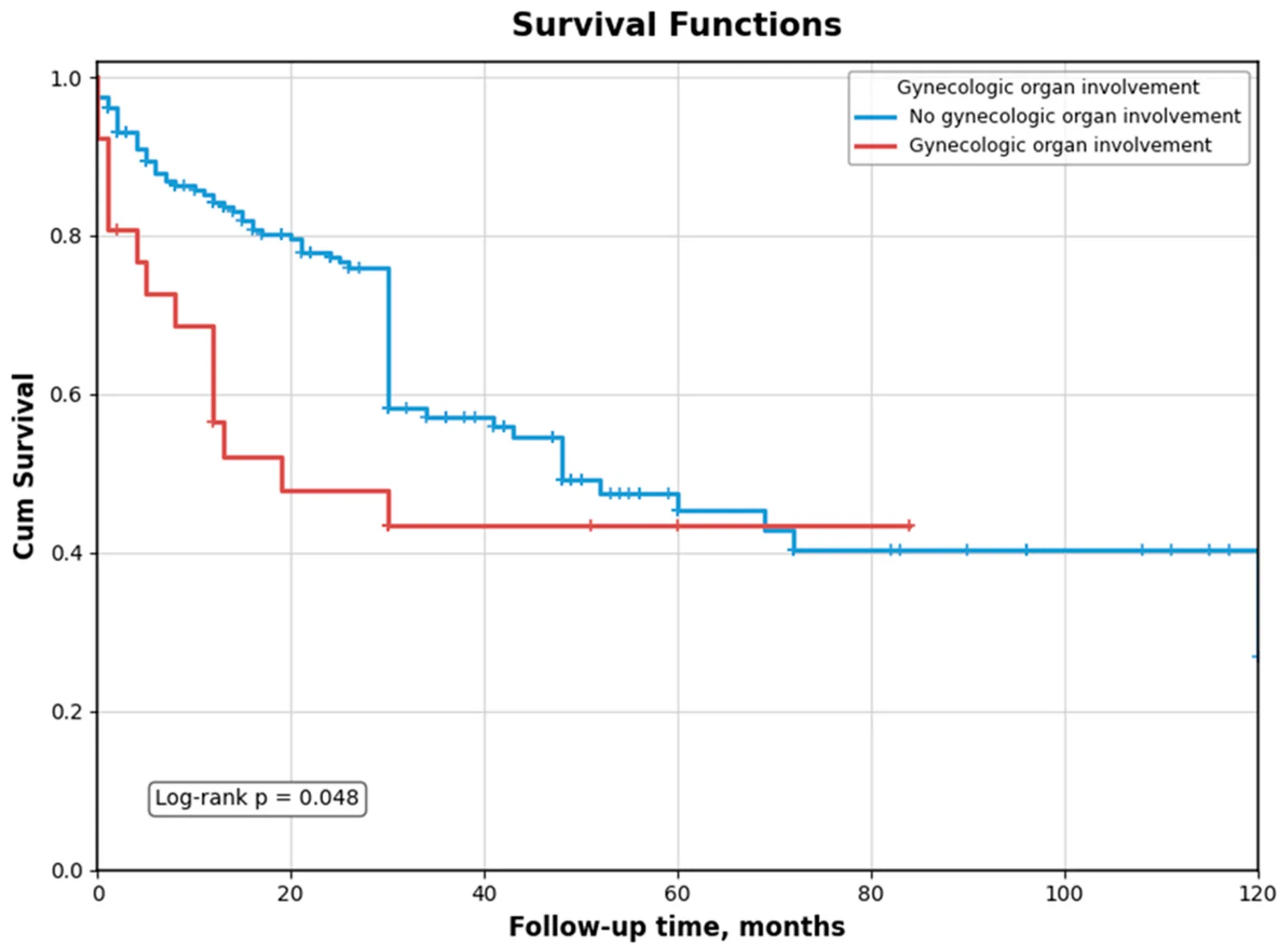
Kaplan–Meier survival curves according to pathological gynecologic organ involvement. Overall survival was significantly shorter in patients with pathological gynecologic organ involvement compared with those without involvement (log-rank *p* = 0.048).

**Figure 4:**
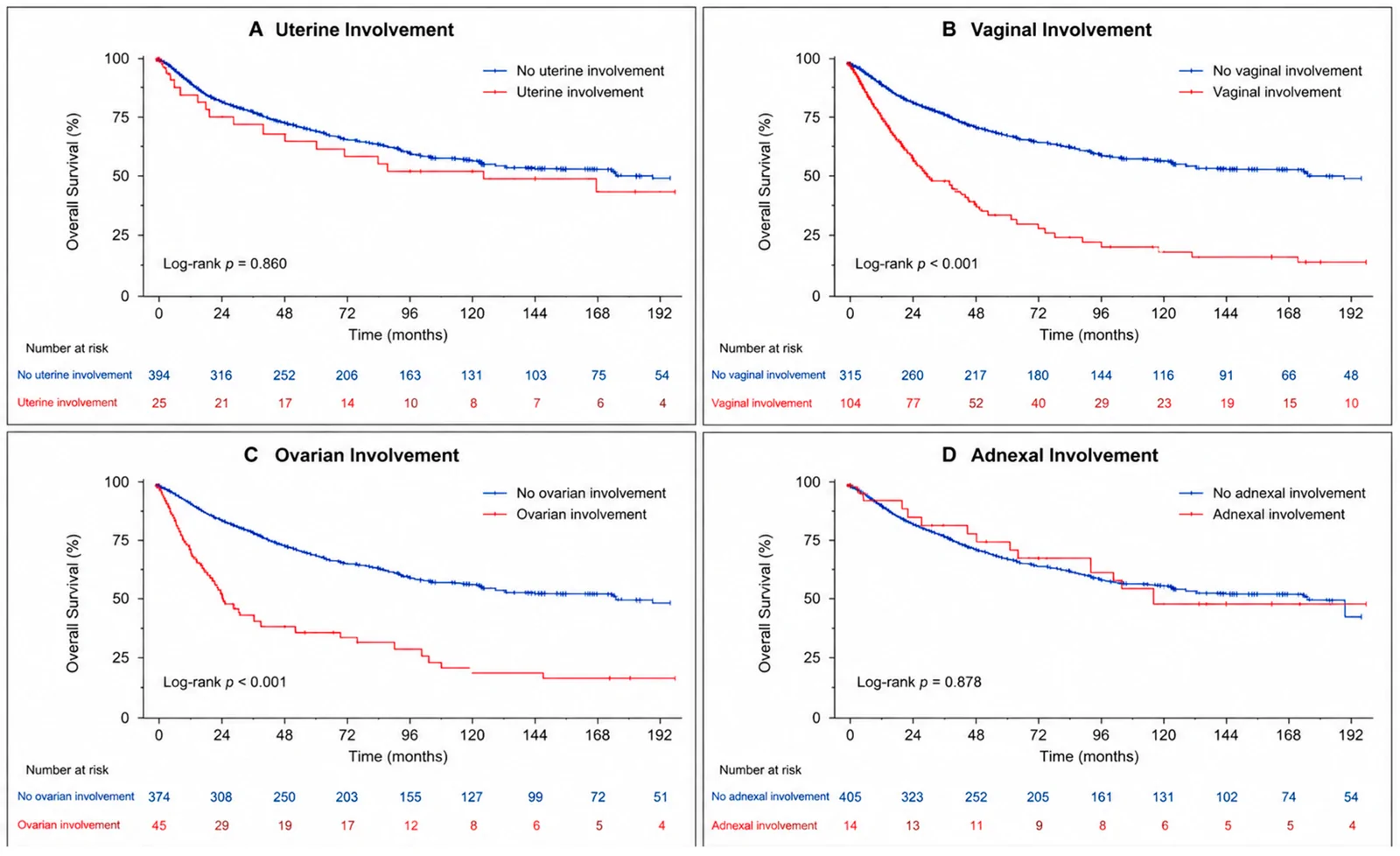
Kaplan–Meier overall survival curves according to organ-specific gynecologic involvement. Separate panels show (**A**) uterine, (**B**) vaginal, (**C**) ovarian, and (**D**) adnexal involvement, each compared with the absence of involvement of the corresponding organ. Log-rank *p*-values were 0.860, <0.001, <0.001, and 0.878, respectively. Because individual patients could have involvement of more than one gynecologic organ, organ-specific analyses across panels were not mutually exclusive.

The survival analyses included 231 patients after excluding one patient with missing follow-up data. Among these patients, OS was significantly shorter in patients with any pathological gynecologic organ involvement than in those without involvement (median OS: 19.0 vs. 48.0 months) (Table 4 and Figure 3). Any pathological gynecologic organ involvement remained associated with an increased risk of mortality after adjustment for the selected covariates in the multivariate Cox regression model (adjusted HR = 1.90; 95% CI: 1.07–3.38; *p* = 0.028) (Supplementary Table S5). In the organ-specific analyses, shorter OS was observed in patients with vaginal (*n* = 11; eight deaths) and ovarian (*n* = 5; four deaths) involvement in unadjusted log-rank analysis (both *p* < 0.001); however, these findings should be interpreted cautiously given the very small subgroup sizes (Table 4 and Figure 4). Overall survival also differed significantly across the mutually exclusive pathological gynecologic organ involvement groups (*p* = 0.002). In the Bonferroni-adjusted pairwise comparison, uterine–vaginal involvement was associated with significantly poorer survival than no pathological gynecologic organ involvement (*p* = 0.002) (Supplementary Table S6).

The median follow-up duration was 30 months (IQR: 12–36 months), and the median time to recurrence was 14 months (IQR: 6–22 months).

## 4. Discussion

In this study, we evaluated the impact of pathological gynecologic organ involvement on survival outcomes in women undergoing RC for bladder cancer and examined the implications for selective organ preservation. The main finding of this study was that pathological gynecologic organ involvement was uncommon, occurring in approximately 11% of patients, and was associated with worse survival. However, pathological gynecologic organ involvement was also strongly associated with advanced pathological stage, particularly T4 disease. Therefore, the observed survival disadvantage may at least partly reflect greater underlying tumor burden and locally advanced disease rather than a direct prognostic effect of pathological gynecologic organ involvement. In the organ-specific analyses, vaginal and ovarian involvement were associated with significantly shorter OS, whereas uterine and adnexal involvement were not significantly associated with OS. However, given the small size of the ovarian subgroup (*n* = 5), this finding should be interpreted cautiously. These findings may contribute to preoperative risk stratification and patient selection for consideration of organ-preserving approaches. However, our study evaluated histopathological organ involvement rather than the oncologic safety of organ preservation. The absence of organ involvement should not, therefore, be interpreted as direct evidence that preservation is oncologically safe.

Our findings add to the growing body of evidence supporting a risk-adapted approach to the management of gynecologic organs during RC. Although pathological gynecologic organ involvement was relatively uncommon, its prognostic impact varied according to the organ involved. Vaginal and ovarian involvement were associated with significantly shorter OS, whereas uterine and adnexal involvement were not significantly associated with OS. Previous evidence has also highlighted the prognostic importance of adverse pathological features, including tumor grade, lymphovascular invasion, and lymph node involvement, in bladder cancer [21]. Nevertheless, the very small number of patients with ovarian involvement (*n* = 5) limits the precision of this estimate and warrants cautious interpretation.

The estimated CSS and RFS probabilities at 60 months were 40% and 60%, respectively. Because OS, CSS, and RFS represent distinct but correlated endpoints within the same cohort, these survival outcomes were evaluated separately, and no formal statistical comparisons between them were performed. Accordingly, the observed differences in survival probabilities should be interpreted descriptively.

In their study, Ali-El-Dein et al. [22] reported that gynecologic organs were infrequently involved in RC specimens, particularly in cases without advanced disease or high-grade histology. Although previous studies have emphasized the rarity of ovarian involvement, our organ-specific analysis showed significantly shorter OS among patients with ovarian involvement. However, this finding was based on only five patients and should, therefore, be interpreted cautiously. Accordingly, the ovarian and other organ-specific survival analyses should be considered exploratory. The aforementioned authors advocated for selective gynecologic organ resection, suggesting that routine removal may not be necessary in the absence of clear tumor invasion. Similarly, by potentially preserving these organs in lower-risk patients, particularly younger women, the opportunity arises to maintain hormonal balance, which can improve postoperative recovery and quality of life, particularly concerning sexual and urinary function [22]. Taylor et al.’s findings [19] further corroborate the low rate of ovarian involvement in RC specimens, emphasizing its rarity and association with advanced stages of the disease and LVI. These observations may be relevant when considering ovarian preservation in carefully selected younger patients or those with less advanced disease; however, this study did not establish the feasibility or oncologic safety of ovarian preservation. Potential hormonal benefits should, therefore, be weighed against oncologic factors individually [19]. Studies by Patel et al. [3] support the feasibility of organ-sparing cystectomy, even in patients with variant histology or locally advanced disease. They concluded that gynecologic organ preservation did not adversely affect survival outcomes in appropriately selected patients. In the context of our data, these previous findings support further investigation of whether organ-specific pathological risk may contribute to patient selection for organ-preserving approaches. However, our study does not provide evidence that preservation based on organ-specific involvement patterns improves quality of life or maintains oncologic control. Additionally, a meta-analysis by Zhang et al. [21] reported that several prognostic factors significantly influenced CSS in BC, including tumor grade, LVI, and lymph node involvement. Similarly to our findings, these results suggest that, while pathological gynecologic organ involvement should be considered, more weight should be placed on the aforementioned prognostic markers while planning RC. This perspective further supports a more individualized approach, in which the necessity of organ resection is weighed against factors directly influencing CSS, particularly in younger patients, where fertility preservation or hormonal considerations might influence the decision [21]. Our findings suggest that the absence of uterine or vaginal involvement may help inform preoperative risk stratification when organ preservation is being considered. Previous studies, including Laukhtina et al.’s [23], have discussed selective organ-preserving approaches in appropriately selected patients; however, our pathological findings do not establish the feasibility or oncologic safety of such an approach. In younger women, potential fertility and hormonal benefits of preserving the ovaries and Fallopian tubes have been discussed [24]; however, the absence or rarity of pathological involvement does not establish the oncologic safety of preserving these organs. Whether such benefits can be achieved without compromising oncologic outcomes requires direct evaluation in appropriately designed prospective studies.

The relatively low rate of neoadjuvant chemotherapy (NAC) use in our cohort (11.2%) should be interpreted in the context of the long study period (2004–2023). The relatively low overall NAC rate may also reflect the inclusion of 50 patients with BCG-unresponsive or very-high-risk Ta/T1 NMIBC, for whom NAC was not routinely indicated, as well as interinstitutional variation in treatment practices. NAC utilization in MIBC has historically been limited and has increased substantially over time; a large national cohort demonstrated an increase in NAC use from 9.7% in 2006 to 32.2% in 2014 [25]. Current clinical guidelines recommend cisplatin-based NAC before radical cystectomy for eligible patients with MIBC [2]. Importantly, NAC may result in pathological downstaging and may, therefore, influence pathological stage and the observed frequency of pathological gynecologic organ involvement at cystectomy. In our cohort, however, NAC was not significantly associated with pathological gynecologic organ involvement in the multivariable analysis (adjusted OR = 0.61, 95% CI: 0.38–2.17; *p* = 0.597). Nevertheless, given the relatively small proportion of patients who received NAC and the long inclusion period, the potential influence of treatment-era differences and NAC-related downstaging should be considered when interpreting the pathological findings [11]. We performed calendar-era sensitivity analyses to explore potential temporal heterogeneity further. Neither categorical treatment era (2004–2013 vs. 2014–2023) nor calendar time modeled continuously was significantly associated with pathological gynecologic organ involvement. Although minimally invasive RC was numerically more frequent in the later era, this difference was not statistically significant in the full cohort. These findings provide some reassurance regarding the temporal stability of the primary pathological outcome; nevertheless, residual confounding related to evolving imaging, pathological classification, systemic treatment, surgical techniques, and follow-up practices cannot be excluded.

Recent studies have further emphasized the importance of preoperative risk stratification in bladder cancer. Mahmoudnejad et al. identified clinical T stage, tumor location, tumor size, concomitant CIS, lymphovascular invasion, and preoperative hydronephrosis as predictors of gynecologic organ involvement in women with MIBC, supporting the use of routinely available preoperative variables for surgical planning [20]. More broadly, quantitative and mathematical modeling approaches have also been applied to bladder cancer treatment, including models evaluating BCG/IL-2 immunotherapy and mitomycin-C treatment dynamics [26,27].

This study has several important strengths. To the best of our knowledge, it represents one of the largest multicenter retrospective cohorts evaluating pathological gynecologic organ involvement in women undergoing RC for bladder cancer. The inclusion of patients from 13 high-volume tertiary referral centers enhances the external validity and generalizability of the findings while reflecting contemporary real-world clinical practice. In addition to evaluating oncologic outcomes, our study provides an organ-specific assessment of gynecologic involvement, demonstrating distinct prognostic implications according to the involved organ. Furthermore, we developed and internally validated preoperative prediction models for pathological gynecologic organ involvement using routinely available clinical variables, providing a preliminary assessment of their potential for preoperative risk stratification. External validation and decision curve analysis are required before clinical implementation. The evaluation of discrimination, calibration, and internal optimism correction provides an initial assessment of model performance; however, clinical utility and net benefit were not directly evaluated. The Youden-derived threshold was used for descriptive assessment of model performance and should not be interpreted as a clinical threshold for selecting patients for organ preservation. At this threshold, the relatively low specificity of Model 2 (45.6%) illustrates the trade-off between minimizing missed gynecologic involvement and avoiding unnecessary organ removal. Although the NPV of 97.2% should be interpreted in the context of the relatively low prevalence of pathological gynecologic organ involvement (11.2%), it represents an absolute improvement of 8.4 percentage points over the overall probability of absence of involvement in the full cohort. Therefore, the model should be considered a risk stratification aid rather than a stand-alone criterion for organ-preserving surgery.

Several limitations should be acknowledged. First, the retrospective design is inherently subject to selection bias and residual confounding, precluding causal inference. This is particularly relevant because the Cox model did not comprehensively adjust for postoperative pathological factors such as pathological T/N stage, margin status, LVI, and variant histology, and pathological gynecologic organ involvement was strongly associated with advanced pathological stage. Therefore, residual confounding by disease severity remains possible, and the observed association between pathological gynecologic organ involvement and poorer survival should not be interpreted as demonstrating that organ involvement itself independently causes worse survival. Second, despite the multicenter design, treatment strategies were not fully standardized across participating institutions. In particular, the use of neoadjuvant chemotherapy, surgical approach, lymph node dissection, and perioperative management varied among centers and may have influenced pathological findings and survival outcomes. Center-level clustering was also not explicitly modeled because the limited number of pathological gynecologic organ involvement events across 13 centers resulted in sparse within-center event counts and could have produced unstable mixed-effects or cluster-robust estimates. Therefore, residual center-level correlation cannot be completely excluded. Third, pathological assessment was performed locally without centralized pathological review, introducing the possibility of interobserver variability in the evaluation of pathological gynecologic organ involvement and variant histology. Fourth, although this study included a relatively large overall cohort, the number of patients with involvement of specific gynecologic organs remained limited, reducing the statistical power of organ-specific subgroup analyses and widening CIs for some estimates. The limited number of pathological gynecologic organ involvement events (*n* = 26) also constrained model complexity. Although Model 2 (EPV = 8.7) was retained as the primary parsimonious prediction model, Model 3 (EPV = 5.2) and the eight-variable multivariable analysis presented in Table 3 (EPV = 3.25) remain susceptible to overfitting and coefficient instability. Therefore, findings from these more complex models should be considered exploratory and interpreted cautiously. Importantly, this study was not designed to directly compare organ-preserving RC with standard radical cystectomy. Therefore, the absence of pathological gynecologic organ involvement cannot by itself establish the oncologic safety of preservation. Such an assessment would require consideration of surgical margin status, occult disease, recurrence patterns, and long-term cancer-specific outcomes in patients who actually undergo organ-preserving surgery [10,16,17,18]. Accordingly, our findings should primarily be viewed as providing information for risk stratification and hypothesis generation rather than as definitive evidence supporting organ preservation. Finally, external validation of the proposed prediction models was not performed; therefore, their clinical applicability should be confirmed in independent prospective cohorts before routine implementation.

## 5. Conclusions

In organ-specific analyses, pathological vaginal and ovarian involvement were associated with significantly shorter OS, whereas pathological uterine and adnexal involvement were not significantly associated with OS. Given the small number of organ-specific involvement events, particularly ovarian involvement, these findings should be interpreted cautiously. The identified preoperative factors may help define patients who could be considered for prospective evaluation of organ-preserving RC; however, the proposed prediction model requires external validation before it can inform surgical decision-making. The feasibility and oncologic safety of preserving specific gynecologic organs cannot be established from the present study and require prospective evaluation. As these findings are exploratory, further prospective studies are warranted to validate these findings and establish evidence-based criteria for selective gynecologic organ preservation during RC.

## Figures and Tables

**Table 1:** Clinicopathological characteristics according to pathological gynecologic organ involvement in women undergoing female radical cystectomy.

Characteristic	No Pathological Gynecologic Organ Involvement	Pathological Gynecologic Organ Involvement	*p*-Value
Age, years	63.85 ± 10.0	64.62 ± 9.25	0.545 *
Smoking status, *n* (%)			0.473 **^†^**
Non-smoker	120 (91.6)	11 (8.4)	
Smoker	86 (85.1)	15 (14.9)	
Comorbidity status, *n* (%)			0.292 **^†^**
None	94 (86.2)	15 (13.8)	
Present	112 (91.1)	11 (8.9)	
Preoperative TUR pathology, *n* (%)			0.600 **^‡^**
Ta	92 (91.1)	9 (8.9)	
T1	51 (86.4)	8 (13.6)	
T2	63 (87.5)	9 (12.5)	
Final cystectomy pathologic T stage, *n* (%)			0.001 ^‡^
T0	21 (84.0)	4 (16.0)	
Ta	11 (91.7)	1 (8.3)	
T1	26 (96.3)	1 (3.7)	
T2	43 (89.6)	5 (10.4)	
T3	87 (98.9)	1 (1.1)	
T4	18 (56.2)	14 (43.8)	
Pathologic N stage, *n* (%)			0.032 ^‡^
N0	147 (87.5)	21 (12.5)	
N1	29 (96.7)	1 (3.3)	
N2	23 (88.5)	3 (11.5)	
N3	3 (75.0)	1 (25.0)	
Nx	4 (100.0)	0 (0.0)	
Variant histology, *n* (%)			0.237 ^‡^
No variant histology	109 (92.4)	9 (7.6)	
Squamous differentiation	60 (87.0)	9 (13.0)	
Sarcomatoid variant	8 (66.7)	4 (33.3)	
Micropapillary variant	8 (100.0)	0 (0.0)	
Glandular differentiation	10 (71.4)	4 (28.6)	
Plasmacytoid variant	8 (100.0)	0 (0.0)	
Lymphoepithelioma-like variant	3 (100.0)	0 (0.0)	
Perineural invasion, *n* (%)			0.985 ^†^
Absent	109 (88.6)	14 (11.4)	
Present	97 (89.0)	12 (11.0)	
Lymphovascular invasion, *n* (%)			0.135 ^‡^
Absent	145 (91.2)	14 (8.8)	
Present	61 (83.6)	12 (16.4)	
Neoadjuvant chemotherapy, *n* (%)			0.441 ^‡^
No	181 (87.9)	25 (12.1)	
Yes	25 (96.2)	1 (3.8)	
Surgical approach, *n* (%)			0.178 ^‡^
Open surgery	190 (89.6)	22 (10.4)	
Laparoscopic/robot-assisted surgery	16 (80.0)	4 (20.0)	

* Student’s *t*-test; ^†^ Pearson’s chi-square test; ^‡^ Fisher’s exact test. Percentages are presented as row percentages. Data were complete for all variables shown (*N* = 232).

**Table 2:** Performance and diagnostic characteristics of preoperative prediction models for pathological gynecologic organ involvement.

Model	N	Events	EPV	AUC (95% CI)	Optimism- Corrected AUC	Brier Score	Calibration Slope	Hosmer–Lemeshow P	Max VIF	Cut-Off	Sensitivity	Specificity	PPV	NPV
Model 1 *	232	26	13.0	0.781 (0.692–0.870)	0.762	0.087	0.94	0.641	1.08	0.161	0.308	0.801	0.163	0.902
Model 2 **	232	26	8.7	0.898 (0.810–0.986)	0.872	0.061	0.91	0.734	1.15	0.108	0.692	0.456	0.138	0.922
Model 3 ***	232	26	5.2	0.913 (0.836–0.990)	0.861	0.058	0.78	0.482	1.42	0.113	0.692	0.558	0.165	0.935

Given the limited number of outcome events (*n* = 26), Model 3 should be interpreted cautiously because of its lower EPV and increased potential for overfitting. Diagnostic characteristics in Table 2 were calculated using the Youden-derived cut-off; the clinically oriented risk-score = 0 definition and its corresponding NPV are presented separately in Supplementary Table S3. * Model 1 included TUR pathology T2 (reference: Ta/T1) and tumor size > 3 cm. ** Model 2 included TUR pathology T2 (reference: Ta/T1), tumor size > 3 cm, and preoperative hydronephrosis. *** Model 3 included TUR pathology T2 (reference: Ta/T1), tumor size > 3 cm, preoperative hydronephrosis, multifocal tumor, and variant histology in TUR specimen. Abbreviations: AUC, area under the receiver operating characteristic curve; CI, confidence interval; EPV, events per variable; VIF, variance inflation factor; PPV, positive predictive value; NPV, negative predictive value; TUR, transurethral resection. Model 2 was selected as the primary preoperative prediction model because it exhibited the most balanced performance across discrimination, optimism-corrected AUC, calibration, events-per-variable ratio, and multicollinearity. Model 3 was considered an extended exploratory model.

**Table 3:** Exploratory univariable and multivariable logistic regression analyses of pathological gynecologic organ involvement.

Variable	Univariable OR (95% CI)	*p*-Value	Adjusted OR (95% CI)	*p*-Value
Age, years	1.02 (0.98–1.06)	0.346	1.01 (0.94–1.09)	0.473
BMI, kg/m^2^	1.08 (0.98–1.19)	0.126	1.06 (0.95–1.18)	0.286
TUR pathology T2 (reference: Ta/T1)	3.12 (0.66–14.86)	0.153	2.74 (1.10–6.84)	0.027
Tumor size > 3 cm	4.48 (1.52–13.21)	0.006	3.96 (1.12–11.77)	0.022
Multifocal tumor	3.26 (1.13–9.42)	0.029	2.63 (1.01–7.56)	0.035
Preoperative hydronephrosis	3.71 (1.58–8.73)	0.003	3.18 (1.25–8.09)	0.017
Variant histology in TUR specimen	3.94 (1.45–10.72)	0.007	3.42 (1.14–10.27)	0.028
Neoadjuvant chemotherapy	0.68 (0.29–1.89)	0.326	0.61 (0.38–2.17)	0.597

The multivariable model included eight predictors and 26 outcome events (EPV = 3.25); therefore, adjusted estimates should be considered exploratory and interpreted cautiously because of the potential for overfitting. Multivariable model included age, BMI, TUR pathology, tumor size, multifocal tumor, preoperative hydronephrosis, variant histology in TUR specimen, and neoadjuvant chemotherapy. Abbreviations: OR, odds ratio; CI, confidence interval; TUR, transurethral resection; BMI, body mass index.

**Table 4:** Kaplan–Meier analysis of overall survival according to pathological gynecologic organ involvement and organ-specific involvement.

Variable	No Involvement, *n*/Deaths	Involvement, *n*/Deaths	Median OS, Months—No Involvement	Median OS, Months—Involvement	Log-Rank χ^2^	*p*-Value
Any pathological gynecologic organ involvement	205/84	26/14	48.0	19.0	3.926	0.048
Uterine involvement	216/92	15/6	48.0	NR	0.031	0.860
Vaginal involvement	220/90	11/8	48.0	12.0	15.969	<0.001
Ovarian involvement	226/94	5/4	48.0	12.0	16.152	<0.001
Adnexal involvement	210/90	21/8	48.0	30.0	0.024	0.878

Abbreviations: OS, overall survival; NR, not reached. Table Notes: Survival time was calculated from radical cystectomy to death or last follow-up. One patient was excluded from the survival analyses due to missing follow-up data (n=231). Comparisons between groups were performed using the log-rank test. Median OS is reported in months. Median OS was not reached (NR) in the uterine involvement group because the survival probability did not fall below 50% during follow-up. Footnote: Denominators in this table refer to the survival analysis cohort (n=231), whereas organ-specific pathological involvement frequencies were calculated using the full analytic cohort (n=232).

## Data Availability

The data that support the findings of this study are available from the corresponding author upon reasonable request.

## Supplementary Materials

The following supporting information can be downloaded at: https://www.mdpi.com/article/10.3390/medicina62081609/s1, Table S1: Calendar-era sensitivity analysis of pathological gynecologic organ involvement and selected treatment characteristics; Table S2. Regression coefficients for the primary preoperative prediction model (Model 2); Table S3: Negative predictive value of low-risk definitions for pathological gynecologic organ involvement; Table S4: Firth penalized logistic regression sensitivity analysis for pathological gynecologic organ involvement; Table S5: Univariable/multivariable Cox proportional hazards regression analyses according to pathological gynecologic organ involvement; Table S6: Pairwise log-rank comparisons of overall survival among mutually exclusive pathological gynecologic organ involvement groups.
