# Supplementary material for: Outcomes and Predictive Factors for Gynecologic Organ Involvement in Radical Cystectomy for Female Bladder Cancer Patients: A Multicenter Retrospective Cohort Study
Source: Medicina (Kaunas). 2026 Aug 21;62(8):1609. doi: 10.3390/medicina62081609 (PMC13515190; doi:10.3390/medicina62081609)
Supplement: Supplementary file 1 [file medicina-62-01609-s001.zip › Supplementary Table S1.pdf]

Supplementary Table S1. Calendar-era sensitivity analysis of gynecologic organ involvement and selected treatment characteristics

Characteristic / Analysis	2004–2013	2014–2023	Effect estimate / AUC	P value
**Patients included, n**	70	162	—	—
**Pathological gynecologic organ involvement, n (%)**	11 (15.7)	15 (9.3)	OR 0.55 (95% CI 0.24–1.26)	0.180*
**Neoadjuvant chemotherapy, n (%)**	8 (11.4)	18 (11.1)	—	1.000*
**Minimally invasive RC, n (%)**	4 (5.7)	16 (9.9)	—	0.420*
**Calendar era logistic model (2014–2023 vs. 2004–2013)**	—	—	AUC 0.558	0.180‡
**Calendar time, continuous (per 5-year increment)**	—	—	OR 0.93 (95% CI 0.61–1.41); AUC 0.547	0.738‡

Table notes: * Fisher's exact test. ‡ Univariable logistic regression. Abbreviations: OR, odds ratio; CI, confidence interval; AUC, area under the receiver operating characteristic curve; RC, radical cystectomy. All percentages were calculated using the full era-specific denominators.
