# Supplementary material for: Outcomes and Predictive Factors for Gynecologic Organ Involvement in Radical Cystectomy for Female Bladder Cancer Patients: A Multicenter Retrospective Cohort Study
Source: Medicina (Kaunas). 2026 Aug 21;62(8):1609. doi: 10.3390/medicina62081609 (PMC13515190; doi:10.3390/medicina62081609)
Supplement: Supplementary file 1 [file medicina-62-01609-s001.zip › Supplementary Table S2.pdf]

Supplementary Table S2. Regression coefficients for the primary preoperative prediction model (Model 2).

Variable	B	S.E.	Wald	p	Exp(B)
**TUR pathology T2 (vs. Ta/T1)**	1.02	0.42	5.90	0.015	2.77
**Tumor size >3 cm**	1.38	0.50	7.62	0.006	3.97
**Preoperative hydronephrosis**	1.16	0.44	6.95	0.008	3.19
**Constant**	-3.55	0.54	43.20	<0.001	0.029

Footnote: Logit(p) = -3.55 + 1.02(TUR pathology T2) + 1.38(tumor size >3 cm) + 1.16(preoperative hydronephrosis), with binary predictors coded as 1=present and 0=absent.
