# Supplementary material for: Outcomes and Predictive Factors for Gynecologic Organ Involvement in Radical Cystectomy for Female Bladder Cancer Patients: A Multicenter Retrospective Cohort Study
Source: Medicina (Kaunas). 2026 Aug 21;62(8):1609. doi: 10.3390/medicina62081609 (PMC13515190; doi:10.3390/medicina62081609)
Supplement: Supplementary file 1 [file medicina-62-01609-s001.zip › Supplementary Table S3.pdf]

Supplementary Table S3. Negative predictive value of low-risk definitions for gynecologic organ involvement

Model	Low-risk definition	Low-risk patients, n (%)	Gynecologic organ involvement in low-risk group, n	Missed involvement, %	NPV, %	Sensitivity, %
**Model 1**	Risk score 0	68/232 (29.3)	3	11.5	95.6	88.5
**Model 2**	**Risk score 0**	**72/232 (31.0)**	**2**	**7.7**	**97.2**	**92.3**
**Model 3**	Risk score 0	48/232 (20.7)	1	3.8	97.9	96.2

Model definitions:
Model 1 included TUR pathology T2 (reference: Ta/T1) and tumor size >3 cm.
Model 2 included TUR pathology T2 (reference: Ta/T1), tumor size >3 cm, and preoperative hydronephrosis.
Model 3 included TUR pathology T2 (reference: Ta/T1), tumor size >3 cm, preoperative hydronephrosis, multifocal tumor, and variant histology on TUR specimen.
Table note: Low-risk status was defined as a risk score of 0. Negative predictive value was calculated using the low-risk group as the test-negative group. Model 2 was selected as the primary reduced preoperative model because it provided a high NPV while identifying a larger low-risk subgroup with fewer predictors than the extended model.
Abbreviations: NPV, negative predictive value; TUR, transurethral resection.
