# Supplementary material for: Outcomes and Predictive Factors for Gynecologic Organ Involvement in Radical Cystectomy for Female Bladder Cancer Patients: A Multicenter Retrospective Cohort Study
Source: Medicina (Kaunas). 2026 Aug 21;62(8):1609. doi: 10.3390/medicina62081609 (PMC13515190; doi:10.3390/medicina62081609)
Supplement: Supplementary file 1 [file medicina-62-01609-s001.zip › Supplementary Table S4.pdf]

Supplementary Table S4. Firth penalized logistic regression sensitivity analysis for pathological gynecologic organ involvement.

Variable	Conventional adjusted OR	Firth OR	95% CI	p
**Age, years**	1.01	1.01	0.96–1.07	0.64
**BMI, kg/m^2**	1.06	1.05	0.95–1.16	0.34
**TUR pathology T2 (ref. Ta/T1)**	2.74	2.48	1.06–5.92	0.036
**Tumor size >3 cm**	3.96	3.42	1.18–9.86	0.025
**Multifocal tumor**	2.63	2.31	0.98–5.71	0.056
**Preoperative hydronephrosis**	3.18	2.85	1.14–7.19	0.026
**Variant histology on TUR specimen**	3.42	3.01	1.05–8.91	0.041
**Neoadjuvant chemotherapy**	0.61	0.70	0.27–1.79	0.46

Footnote: Firth penalized logistic regression was performed as a sensitivity analysis using the same eight predictors as the conventional multivariable model. Penalized ORs with 95% CIs are presented.
