# Supplementary material for: Outcomes and Predictive Factors for Gynecologic Organ Involvement in Radical Cystectomy for Female Bladder Cancer Patients: A Multicenter Retrospective Cohort Study
Source: Medicina (Kaunas). 2026 Aug 21;62(8):1609. doi: 10.3390/medicina62081609 (PMC13515190; doi:10.3390/medicina62081609)
Supplement: Supplementary file 1 [file medicina-62-01609-s001.zip › Supplementary Table S5.pdf]

Supplementary Table S5a. Univariable Cox proportional hazards regression analyses according to gynecologic organ involvement

Variable	Patients, n	Deaths, n	HR (95% CI)	P value
**Any gynecologic organ involvement**	231	98	1.73 (0.98–3.04)	0.060
**Uterine involvement**	231	98	0.93 (0.41–2.13)	0.865
**Vaginal involvement**	231	98	3.85 (1.85–8.02)	<0.001
**Ovarian involvement**	231	98	6.23 (2.22–17.49)	0.001
**Adnexal involvement**	231	98	1.05 (0.55–2.02)	0.882

Supplementary Table S5b. Multivariable Cox proportional hazards model for overall survival

Variable	Adjusted HR (95% CI)	P value
**Any gynecologic organ involvement**	1.90 (1.07–3.38)	0.028
**Age, years**	1.04 (1.02–1.06)	0.001
**TUR pathology T2 (reference: Ta/T1)**	1.70 (1.11–2.61)	0.015
**Tumor size >3 cm**	0.85 (0.52–1.38)	0.509
**Preoperative hydronephrosis**	0.87 (0.58–1.32)	0.520

Abbreviations: HR, hazard ratio; CI, confidence interval; TUR, transurethral resection.
Table note: Overall survival was calculated from radical cystectomy to death from any cause or last follow-up. One patient was excluded from the survival analyses because of missing follow-up data; therefore, survival analyses included 231 patients. Hazard ratios were estimated using Cox proportional hazards regression models.
