# Supplementary material for: Outcomes and Predictive Factors for Gynecologic Organ Involvement in Radical Cystectomy for Female Bladder Cancer Patients: A Multicenter Retrospective Cohort Study
Source: Medicina (Kaunas). 2026 Aug 21;62(8):1609. doi: 10.3390/medicina62081609 (PMC13515190; doi:10.3390/medicina62081609)
Supplement: Supplementary file 1 [file medicina-62-01609-s001.zip › Supplementary Table S6.pdf]

Supplementary Table S6. Pairwise log-rank comparisons of overall survival among mutually exclusive gynecologic organ involvement groups

Pairwise comparison	Log-rank χ²	Unadjusted P value	Bonferroni-adjusted P value
**No gynecologic organ involvement vs uterine-only involvement**	0.002	0.961	1.000
**No gynecologic organ involvement vs adnexal-only involvement**	3.701	0.054	0.544
**No gynecologic organ involvement vs uterine-vaginal involvement**	14.319	<0.001	0.002
**No gynecologic organ involvement vs multiple gynecologic organ involvement**	0.003	0.953	1.000
**Uterine-only involvement vs adnexal-only involvement**	1.691	0.193	1.000
**Uterine -only involvement vs uterine-vaginal involvement**	2.905	0.088	0.883
**Uterine -only involvement vs multiple gynecologic organ involvement**	0.093	0.760	1.000
**Adnexal-only involvement vs uterine-vaginal involvement**	0.017	0.896	1.000
**Adnexal-only involvement vs multiple gynecologic organ involvement**	1.365	0.243	1.000
**Uterine -vaginal involvement vs multiple gynecologic organ involvement**	1.456	0.228	1.000

Table note: Pairwise comparisons were performed using the log-rank test. Bonferroni adjustment was applied to account for multiple comparisons. The only pairwise comparison that remained statistically significant after adjustment was no gynecologic organ involvement versus uterine-vaginal involvement. Abbreviations: χ², chi-square.
